# Electrophysiological correlates of morphological processing in Chinese compound word recognition

**DOI:** 10.3389/fnhum.2013.00601

**Published:** 2013-09-24

**Authors:** Yingchun Du, Weiping Hu, Zhuo Fang, John X. Zhang

**Affiliations:** ^1^Key Laboratory of Behavioral Science, Institute of Psychology, Chinese Academy of Sciences, Beijing, China; ^2^MOE Key Laboratory of Modern Teaching Technology, Shaanxi Normal University, Xi'an, China; ^3^Department of Psychology, Sun Yat-Sen University, Guangzhou, China; ^4^Department of Psychology, Fudan University, Shanghai, China

**Keywords:** morphological processing, compound word, delayed repetition, morpheme, Chinese

## Abstract

The present study investigated the electrophysiological correlates of morphological processing in Chinese compound word reading using a delayed repetition priming paradigm. Participants were asked to passively view lists of two-character compound words containing prime-target pairs separated by a few items. In a *Whole Word* repetition condition, the prime and target were the same real words (e.g., 
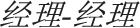
, manager-manager). In a *Constituent* repetition condition, the prime and target were swapped in terms of their constituent position (e.g., 
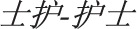
, the former is a pseudo-word and the later means *nurse*). Two ERP components including N200 and N400 showed repetition effects. The N200 showed a negative shift upon repetition in the *Whole Word* condition but this effect was delayed for the *Constituent* condition. The N400 showed comparable amplitude reduction across the two priming conditions. The results reveal different aspects of morphological processing with an early stage associated with N200 and a late stage with N400. There was also a possibility that the N200 effect reflect general cognitive processing, i.e., the detection of low-probability stimuli.

## Introduction

Understanding to the nature of morphological processing is critical to the investigation of how written words are represented in the brain. Over the past three decades, morphological processing has been central to the study of mental lexicon (Libben and Jarema, 2004). One key issue about morphological processing is whether morphemically complex words are represented in mental lexicon as a whole, or by their constituent morphemes (Chen and Chen, 2006). In the early literature, a strong view referred to as the full-listing models proposed that complex words including compound words are processed as a single unit with no reference to its constituents (e.g., Butterworth, 1983; Bybee, 1995). However, much evidence has accumulated supporting a decompositional view where complex words are processed by access to and combination of their constituent morpheme representations (e.g., Taft and Forster, 1975; Libben et al., 1999; McKinnon et al., 2003). As a compromise, many recent models on morphological processing combine features from both models (Caramazza et al., 1988; Schreuder and Baayen, 1995; Baayen et al., 1997; Isel et al., 2003). There is also evidence that interplays between factors such as frequency (Alegre and Gordon, 1999), morphological type (Miceli and Caramazza, 1988), and language background of a speaker (Portin et al., 2008) determine whether a multimorphemic word is stored and recognized as a full form or via decomposition during lexical-semantic processing.

As one major way to produce morphologically complex words, compounding has been widely attested in many languages. It provides a unique opportunity for assessing the combinatorial mechanisms inherent to language production and comprehension (Badecker, 2007). It is particularly important for some languages such as Chinese where more than 70% of the vocabulary is made up of multiple-character compound words. Compared with the other two types of morphological complex words, i.e., inflectional words (e.g., *depart—departing*) and derivational words (e.g., *agree-agreement*), which are prevalent in most alphabetic languages, there has been relatively less research in compound word recognition.

Morphological processing is clearly important for both inflectional and derivational words, especially irregular words. However, whether this is also the case for compound words is less clear, as morphemes in compound words are relatively separable and easy to identify (Chung et al., 2010). On the one hand, morphological decomposition seems to offer a very effective route for compound word comprehension. On the other hand, compounds are sensitive to semantic drift and thus frequently show high degrees of semantic opacity that would thwart a routine morphological decomposition (Libben, 1998).

Early evidence for morphological decomposition of compound words mainly came from behavioral studies showing that constituent frequencies affected lexical decision times of compound words (e.g., Zhang and Peng, 1992; Zhou and Marslen-Wilson, 1994), or from the examination of morphological priming (Zhang and Peng, 1992; Liu and Peng, 1997). Nowadays, electrophysiological techniques with high temporal resolution have been used to investigate the neural dynamics of morphological processing. Due to differences in word types and tasks in individual studies, the evidence about the neural correlates of morphological processing is far from consistent. For example, with auditory stimuli, Koester et al. (2004) found gender violations of initial constituents resulted in a left-anterior negativity (LAN) for both opaque and transparent compounds, which was interpreted as an index of morphosyntactic decomposition. Vergara-Martínez et al. (2009) revealed that high-frequency first constituents elicited larger negativities starting in the 100–300 ms time window, while low-frequency second constituents elicited larger N400 amplitudes than high-frequency second constituents. Chiarelli et al. (2007) found larger LAN and N400 for compound than non-compound words.

In a series of experiments using an immediate repetition paradigm, we found that both full (
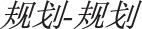
, plan—plan) and partial morphological (
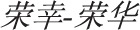
, honor—nabobism) overlap between two-constituent prime-target pairs (both being real words) elicited an enhanced N200 response and a reduced N400 response (Zhang et al., 2012; Jia et al., 2013). Further, the N200 enhancement effect was larger when the prime and target words were fully overlapped compared with partially overlapped.

Although these results indicate that both N200 and N400 are modulated by morphological similarity, it is unclear whether they are truly the electrophysiological correlates of morphological processing. This is because words sharing morphological elements are usually also related in form, phonology, and meaning resulting in priming effect in N200 and N400. In addition, participants may adopt specific response strategies in the immediate repetition paradigm producing priming effects that would otherwise be absent in normal reading (Bozic et al., 2007).

To avoid these problems, the present study turned to a delayed repetition priming paradigm, which has been well-established to be a powerful tool to investigate the nature of internal stimulus representation (see Henson and Rugg, 2003; Schacter et al., 2007 for reviews). In a delayed repetition task, members of a word pair are separated by varying numbers of intervening words, which reduces effects of task strategies. Previous studies using this task have shown that morphological effects are preserved over long lags, whereas semantic and form effects drop away sharply as the number of intervening items increases (Napps, 1989; Bentin and Feldman, 1990; Zwitserlood et al., 2002). More importantly, an implicit task was used to minimize strategic processes due to task demands and to probe reading processes in a more natural way (Vartiainen et al., 2009).

Based on our previous studies (Zhang et al., 2012; Jia et al., 2013), priming effects in the N200 and N400 components would be expected. While N400 has been extensively studied and used as an effective dependent variable for examining many aspect of language processing (for review, see Kutas and Federmeier, 2011). In comparison, the functional significance of N200 is far from clear. The N200, also called N2 in some literature, refers to the second negative wave peaking between 200 and 350 ms after stimulus onset. It is suggested that the N200 elicited by visual stimuli should be divided into at least three subcomponents: a fronto-central (anterior) component related to the detection of novelty or mismatch from a perceptual template when the eliciting stimuli are attended, a second fronto-central component related to cognitive control (encompassing response inhibition, response conflict, and error monitoring), and one or two posterior N2s related to some aspects of visual attention (for review, see Folstein and Van Petten, 2008). The specific pattern of priming effect may help to reveal whether and how these N2 components are related to the N200 identified in our previous word recognition studies (Zhang et al., 2012; Jia et al., 2013).

## Methods

### Participants

Twenty college students (10 males, age range from 19 to 30 years, mean ±SD = 23.3 ± 3.0 years) participated in this experiment with monetary compensation. All were right-handed native speakers of Mandarin Chinese with normal or corrected-to-normal vision. Their handedness scores were between 54 and 100, which were assessed by the Edinburgh Handedness Inventory (Oldfield, 1971). None of them reported any neurological or psychiatric diseases. Informed consent was obtained in accordance with guidelines from the Institute of Psychology, Chinese Academy of Sciences, Beijing, China.

### Stimuli

**Figure 1:**
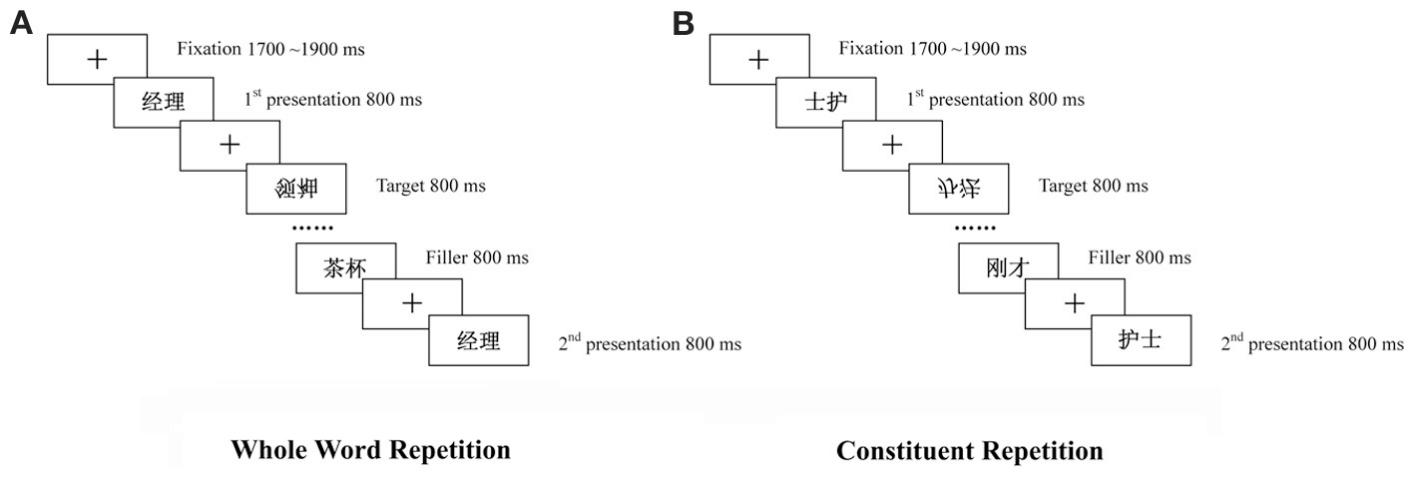
**Illustration of the trial structure in the two repetition conditions.** Participants viewed a list of two-character items to detect rare upside-down target items. **(A)** The *Whole Word* repetition condition involved repetition of the same compound word (i.e., 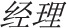 meaning *manager*) across several intervening trials. **(B)** The *Constituent* repetition condition involved presenting a compound word first in its swapped version (i.e., 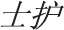 as a nonsense pseudo-word), followed after several intervening items by the word in its normal reading format (i.e., 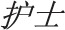 meaning *nurse*).

There were two experimental conditions both involving presenting a prime-target word pair separated by several intervening items (Figure 1). In the *Whole Word* repetition condition, the prime and target were the same real word. In the *Constituent* repetition condition, the prime and target were swapped in terms of their constituent position. In modern Chinese, a compound word is always read from left to right and swapping the characters in position for a two-character word would usually produce a meaningless pseudo-word without pre-existing linguistic representations as a whole [except for a small set of reversible words as studied in Zhang et al. (2004)]. For example, after the swap, the word 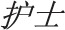
meaning *nurse* became 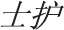 which was not a real word anymore. Also, the pseudo-words were not homophonic to any real word. So, for the *Constituent* repetition condition, the prime was a pseudo-word while the target was a real word but they shared the same two constituents. It should be noticed that the pseudo-words are unfamiliar novel items and novelty may confound with the repetition effect in the *Constituent* condition.

Character combinability was controlled across the two priming conditions as each critical word was assigned to the *Whole Word* condition for half of the participants but to the *Constitute* condition for the other half. The chance of each character appearing in the first or second position of a compound word varies for different characters. However, for high frequency characters as used in the present study, it is mostly the case that they could appear in both the first and the second positions. Therefore, it is very hard or almost impossible for participants to make use of the frequency information to infer whether the word was a real word or not. A pseudo-word was very much like a real word except its specific character combination as a whole unit was not associated with any pre-existing linguistic representations.

The critical stimuli contained a list of 144 two-character words selected from an online corpus based on a research project of Middle Tennessee State University (). The mean stroke number of the words (sum of the two characters) was 15.3 (*SD* = 3.9), and the mean word frequency was 815 occurrence per million (*SD* = 783, frequency range = 55–3476, median = 598), respectively. To reduce explicit attention to stimulus repetitions of the critical words, there were 144 two-character filler items including 60 real words and 84 pseudo-words. The pseudo-words consisted of two Chinese characters matching the critical words in visual complexity (i.e., stroke number). Each pseudo-word as a whole unit was neither a real word nor homophonic to any real word. There were also 60 two-character real words serving as the target items. All the real filler words and the target words were selected from the same online corpus matching the critical words in stimulus characteristics wherever applicable.

### Procedure

Participants were seated in a dimly lit and sound-attenuated room. All visual stimuli were presented on a computer monitor that was about 1 m away from participants' eyes. All word stimuli were displayed at high contrast as black words on a white background, subtending visual angles of 4.3 × 2.3^°^. Participants were instructed to remain relaxed and to refrain from moving throughout the experiment.

Each participant completed 6 blocks, each with 82 trials. The first block served as practice and was not analyzed. As shown in Figure 1, each trial started with a fixation at the screen center for a period jittered between 1700 and 1900 ms. The fixation was then replaced by the central presentation of a visual item that was turned off 800 ms later, followed immediately by the fixation of the next trial. The item could be a critical word, a filler or a target item.

In each block, there were 12 critical words used for *Whole Word* condition, 12 for *Constituent* condition, 10 real filler words, 14 filler pseudo-words, and 10 target words. The different types of items were pseudo-randomly intermixed with the following constraints. Each critical item was presented in two trials separated on average by 4 intervening trials (ranging from 1 to 7 trials or 5.1 to 20.4 s).

Participants responded to the target items by pressing a button as quickly as possible with their right index finger. For non-target items, they should refrain from making any response. The detection task was only to ensure attention to the critical non-target items for which no response was required to minimize motor-related contaminations on the ERPs. Such tasks have been widely used in alphabetic reading studies. For example, in Price et al. (1996) and Jamal et al. (2011) study, participants were instructed to perform a non-linguistic visual feature detection task, i.e., to detect the presence or absence of ascenders within a stimulus.

### Electroencephalogram recording and data analysis

The electroencephalogram (EEG) was recorded from the scalp through 64 non-polarizable Ag/AgCl sintered electrodes in a pre-configured cap. The position of electrodes followed the extended 10–20 convention. The EEG was continuously sampled at a rate of 1000 Hz and bandpass filtered (0.05–100 Hz) using the Neuroscan EEG system (NeuroScan Inc., Compumedics, Australia). Electrode impedance was maintained below 5 kΩ. In addition to the scalp sites, the horizontal EOG was recorded at the outer canthi of both eyes and the vertical EOG was recorded between supraorbit and suborbit of the left eye. The left mastoid was used as the recording reference. Reference was changed offline to the average of the two mastoids.

Eye movement artifacts were removed using regression-based weighting coefficients (Semlitsch et al., 1986). This method subtracted a fraction of an EOG from the EEG channels on a sweep-by-sweep, point-by-point basis. EEG segments were abstracted from 150 ms before stimuli onset to 850 ms post stimuli onset. The 150 ms pre-stimuli period was used as the baseline. The segments were baseline corrected and bandpass filtered (0.5–30 Hz). Segments with amplitude exceeding ±50 μv in any scalp channel were excluded from analysis (less than 2% of trials were rejected). Averaged ERPs were computed separately for the first and second time of presentation of the critical items in the two repetition conditions. The filler items and the targets were not analyzed.

## Results

**Figure 2:**
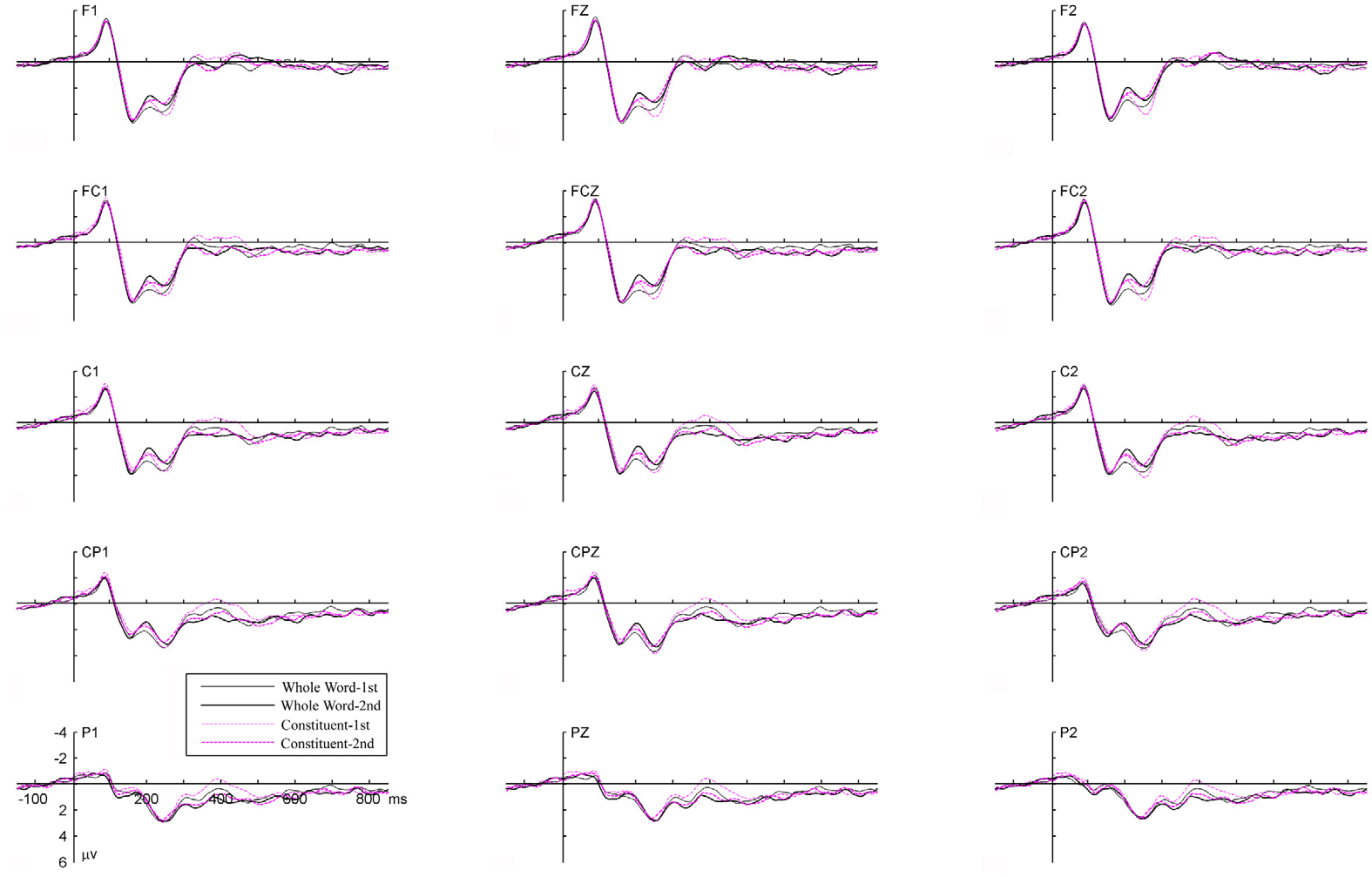
**Grand average ERP waveforms elicited by the four types of critical items in selected electrodes.** Whole Word-1st and Whole Word-2nd indicate the first and second time of presentation in the *Whole Word* repetition condition. Constituent-1st and Constituent-2nd indicate the first and second time of presentation in the *Constituent* repetition condition.

**Figure 3:**
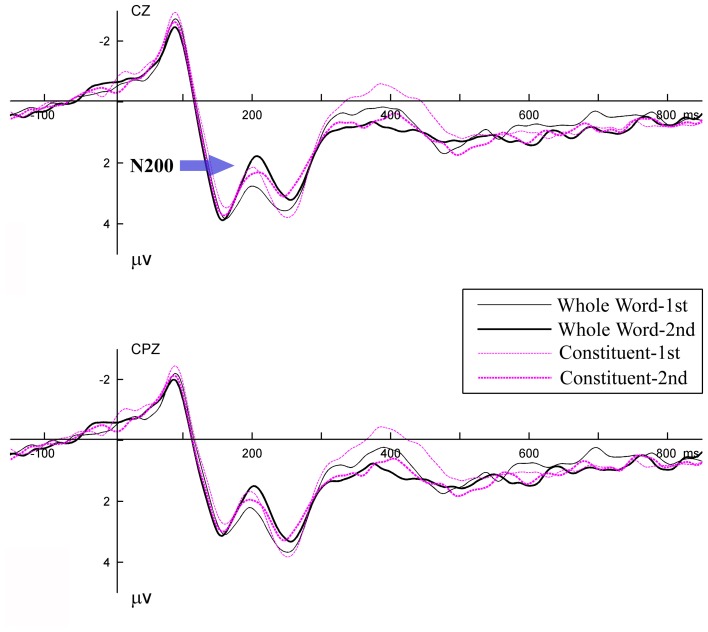
**Two electrodes from Figure 2 highlighted for clarity.** Legends are the same as in Figure 2.

Data from two participants were excluded with exceedingly low target detection accuracy (50 and 62%). For the remaining 18 participants, target detection was both fast and highly accurate with a mean response time of 561 ms (*SD* = 42) and a mean accuracy of 92.8% (*SD* = 7.0). Mean false alarm rate was below 1.5% for all types of non-target items with a mean of 0.5% (*SD* = 1.2). These behavioral results indicate that the included 18 participants followed the instructions and were attentive to the critical items in the detection task. The grand-average waveforms (based on 18 participants) for all experimental conditions are plotted in Figure 2 for 15 representative electrodes. Waveforms at two electrodes Cz and CPz are highlighted in Figure 3 for clarity.

As shown in the figures, both repetition conditions elicited similar N1-P2 complexes regardless of whether the stimuli were in their first presentation or second presentation. A negative deflection was elicited between 200 and 230 ms, with similar latency and distribution as the N200 component reported in our previous studies (Zhang et al., 2012; Jia et al., 2013). Following the N200, there was a positive shift peaking around 260 ms and a broad negativity peaking around 400 ms (N400).

**Figure 4:**
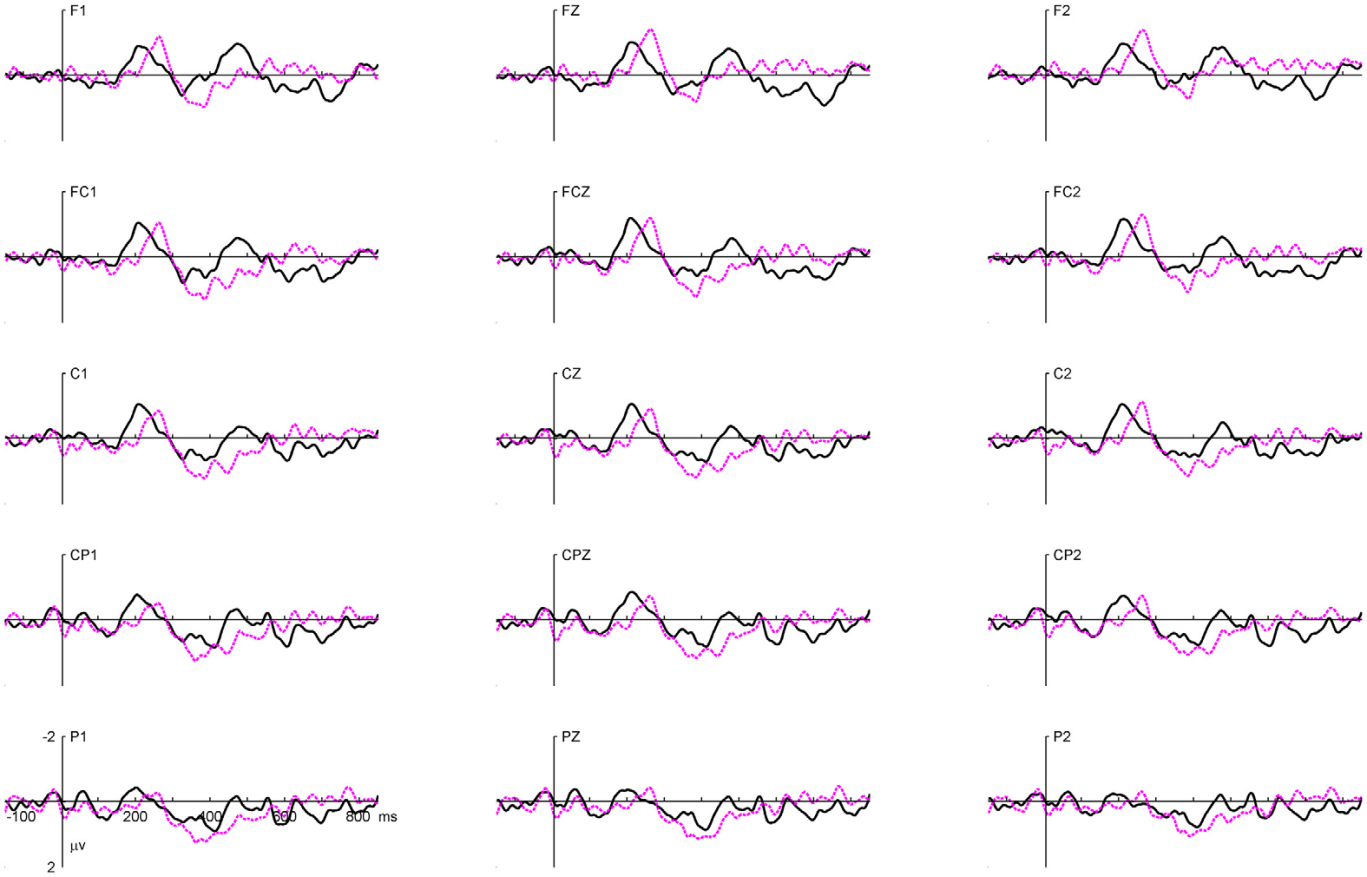
**Grand average difference waveforms at 15 scalp electrode sites for *Whole Word* (black) and *Constituent* (purple) conditions.** Difference waves were computed by subtracting the mean voltage for first presentation from those for second presentation trials.

To further characterize the 260 ms positive shift, difference waves for the two experimental conditions were computed by subtracting the mean voltage for the first presentation trials from those for the second presentation trials (see Figure 4). The difference waves highlight the components of interest more clearly by removing variations in voltage that were common to all conditions. By visual inspection, the difference wave around the 260 ms period under the *Constituent* condition was comparable to that in the N200 interval under the *Whole Word* condition. This indicates that the 260 ms positive shift in the *Constituent* condition was likely a delayed N200 effect.

By visual inspection, the N200 effect and the delayed N200 effect were mainly distributed in the fronto-central region, and the N400 was mainly distributed in the centro-parietal region. Preliminary analysis did not reveal any significant effect of repetition or condition (*Whole Word* vs. *Constituent*) in the N1 and P2 time windows. Four-Way repeated-measures ANOVAs with Geisser-Greenhouse correction were conducted on the averaged amplitudes of the N200 and the delayed N200 effects, with experimental condition, time of presentation (first vs. second), laterality (midline, left hemisphere, and right hemisphere), and electrode position (frontal: FZ, F1/2; fronto-central: FCZ, FC1/2; central: CZ, C1/2) as factors. The same ANOVA was performed on the N400 component except that the electrode position was different (fronto-central: FCZ, FC1/2; central: CZ, C1/2; centro-parietal: CPZ, CP1/2).

The mean amplitude of N200 was computed for the 185–235 ms time window. The main effects were significant for time of presentation [*F*_(1, 17)_ = 11.1, *p* < 0.01], and for electrode position [*F*_(2, 34)_ = 5.9, *p* < 0.01]. The mean amplitude of N200 was significantly increased (more negative) from the first to the second presentation (3.2 vs. 2.7 μ v). The interaction between time of presentation and condition was also significant [*F*_(1, 17)_ = 7.7, *p* < 0.05]. For the *Whole Word* condition, compared with the first presentation, the words' second presentation elicited a more negative going N200 [3.4 vs. 2.6 μ v, *t*_(17)_ = 5.1, *p* < 0.001]. For the *Constituent* condition, the first and second presentations elicited comparable N200 effects (3.0 vs. 2.9, *p* > 0.5). No other interaction was significant.

The mean amplitude of the delayed N200 effect was computed from the 235 to 285 ms time window. The main effects were significant for time of presentation [*F*_(1, 17)_ = 5.4, *p* < 0.05], and for laterality [*F*_(2, 34)_ = 3.3, *p* < 0.05]. The waveform was more positive going for the first presentation than for the second presentations (3.3 vs. 2.8 μ v), and was more positive going on the midline and right hemisphere than on the left hemisphere (3.1 vs. 2.9 μ v, 3.2 vs. 2.9 μ v). The interaction between time of presentation and electrode position was significant [*F*_(2, 34)_ = 7.6, *p* < 0.01].

**Figure 5:**
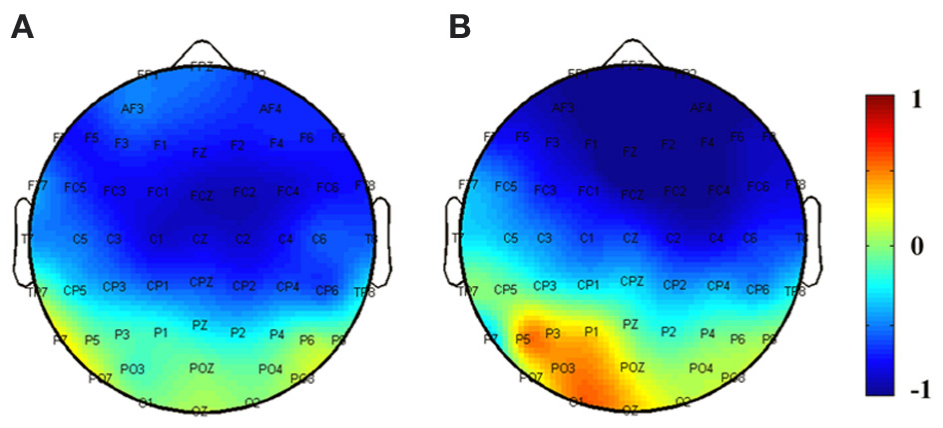
**Topographical maps plotted based on difference waves for: (A) *Whole Word* condition in the N200 effect time window, and (B) *Constituent* condition in the delayed N200 effect time window**.

In addition, to help visualize the distribution of the repetition effect, topographical voltage maps were constructed based on the mean amplitudes of difference waves measured within the N200 and the delayed N200 effects time windows (see Figure 5). A within-subject ANOVA was conducted on the mean amplitude of the difference waves with effect type (the N200 effect vs. the delayed N200 effect) and brain area (anterior, central, and centro-parietal) as factors. The results showed a significant interaction effect [*F*_(2, 34)_ = 5.4, *p* < 0.01]. The delayed N200 effect showed a more anterior distribution [*F*_(2, 34)_ = 8.0, *p* < 0.001], while the N200 effect showed no significant differences across brain regions (*p* > 0.1).

The mean amplitude of N400 was computed from the 330 to 450 ms time window. There were significant main effects for condition [*F*_(1, 17)_ = 7.8, *p* < 0.05], time of presentation [*F*_(1, 17)_ = 18.1, *p* < 0.001], and electrode position [*F*_(2, 34)_ = 4.0, *p* < 0.05]. The N400 was less negative going in the second presentation than in the first presentation (0.8 vs. 0.1 μv), and in the *Whole Word* condition than in the *Constituent* condition (0.7 vs. 0.3 μv). The amplitude reduction across the two presentations was larger for the *Constituent* condition (from 0.7 to −0.2 μv) than for the *Whole Word* condition (from 0.9 to 0.4 μv), although the difference was not significant as interaction between time of presentation and condition was not significant [*F*_(1, 17)_ = 2.2, *p* > 0.1]. The interaction between time of presentation and laterality was significant [*F*_(2, 34)_ = 6.2, *p* < 0.01], with the difference between first and second presentation at the right hemisphere being smaller than that at the midline and left hemispheres (*p*s < 0.05). The interaction between condition and electrode position was also significant [*F*_(2, 34)_ = 3.7, *p* < 0.05], with the difference between conditions at the fronto-central area being smaller than that at the central (*p* < 0.05) and centro-parietal area (although not significant, *p* = 0.06).

## Discussion

In the present study, we combined the recording of ERPs with a delayed repetition priming paradigm to investigate the neural dynamics of morphological processing in Chinese compound word reading. According to previous studies, only morphological priming can be preserved over long lags, whereas semantic, phonological, and form priming drop away sharply as the number of intervening items increases (Napps, 1989; Bentin and Feldman, 1990; Zwitserlood et al., 2002). In the current study, priming effects over long lags were observed in two components including N200 and N400, suggesting their association with morphological processing.

The earliest ERP response sensitive to the manipulation of stimulus repetition was the N200 component. In this time window, the *Whole Word* condition showed an amplitude enhancement (or a negative shift) upon repetition, this effect was delayed for the *Constituent* repetition condition. In the *Whole Word* repetition condition, all word information such as morphological, orthographic, and semantic information were repeated in the second presentation. In comparison, in the *Constituent* repetition condition, only the orthographic and semantic information of the constituent characters were repeated since the primes were not real words. The time difference between the two repetition conditions may indicate facilitated lexical processing at the whole word level as opposed to the constituent level.

One major difference between the N200 effect in the present study and those electrophysiological correlates of morphological processing in earlier literature is the relative early latency of N200. In previous EEG and MEG data on Finnish, English, German, Spanish, and Catalan languages (Penke et al., 1997; Rodriguez-Fornells et al., 2001; Domínguez et al., 2004; Fiorentino and Poeppel, 2007; Vartiainen et al., 2009), no effect of morphology within the first 200 ms following word onset was found. The reason for lacking an early morphology effect in alphabetic language studies may be because their stimuli were usually inflectional or derivational words rather than compound words. There has been evidence that different types of morphologically complex words involve different processing mechanisms. For example, compound words were processed more quickly than matched monomorphemic words (Ji et al., 2011), whereas inflected words were harder to recognize than matched morphologically simple words (Vartiainen et al., 2009).

Another major difference between the present N200 effect and the usually reported repetition effect is that the N200 here showed repetition enhancement instead of repetition suppression. Most studies adopting the neural priming paradigms showed reduced neural responses upon repetition, referred to as neural suppression (e.g., Gauthier, 2000). Furthermore, repetition suppression is generally considered as an effect of stimulus repetition *per se*, occurring independent of other psychological or neurophysiological variables. In contrast, cognitive variables including stimulus recognition, learning and explicit memory can bias repetition effects in BOLD response toward enhancement instead of suppression (for review, see Segaert et al., 2013).

In the electrophysiological literature, the N2 component has been tightly related with the brain function of cognitive control. Studies using the oddball paradigm demonstrated that when targets (go) and non-targets (no-go) were presented with equal probabilities, no-go trials would elicit a larger N2 at frontal scalp site (Ford et al., 1979; Kok, 1986). Czigler et al. (1996) extended this result by showing that low-probability stimuli elicited a larger N2 and the probability effect on the N2 was much larger in the no-go trial than the go trial at the fronto-central scalp site. Bruin and Wijers (2002) further showed that as the probability of no-go stimuli decreased, the N2 elicited by these events increased in amplitude. In the present study, repeated word stimuli (the second presentation) were low-probability non-targets. The enhanced N200 effect elicited by these repeated words may possibly be identified with the larger no-go N2 effect in the above-mentioned oddball paradigm. If this is the case, the N200 may reflect a more general cognitive mechanism as opposed to specific morphological processing. According to the topographical voltage maps, the delayed N200 effect showed a more anterior distribution compared with the N200 effect, possibly reflecting some processes related to novelty detection as the pseudo-words in the *Constituent* condition were unfamiliar items or novel to some degree.

Although pseudo-words are not associated with pre-existing semantic representations, word-like pseudo-words may partially activate the semantic representations of their real-word lexical neighbors (Holcomb et al., 2002). Therefore, the pseudo-word primes in the *Constituent* condition may activate phonologically/orthographically related words and consequently the semantic network, producing the repetition priming effect in N400. Statistical analysis of N400 showed that the priming effect size was comparable across the *Constituent* repetition condition and the *Whole Word* repetition condition, suggesting that the N400 effects reflected primarily morpheme-related processing. Note that the *Constituent* condition seems to involve more extensive semantic processing as shown by the prolonged N400 in this condition, compared with the *Whole Word* condition. In the literature, N400 has been shown to be modulated by morphologically related pairs and the effect size (amount of amplitude reduction) is dependent on the strength of the relation (Domínguez et al., 2004; Lavric et al., 2007). The present finding that N400 is associated with morphological processing is consistent with such previous results.

One more general question about morphology is whether it is a discrete and independent element of lexical structure or it simply emerges from the convergence of form and meaning. In an fMRI study of morphological processing in English, brain regions sensitive to morphological structure overlapped almost entirely with regions sensitive to orthographic (left occipito-temporal cortex) and semantic relatedness (left middle temporal gyrus), suggesting that morphology emerges from the convergence of form and meaning (Devlin et al., 2004). Therefore, morphology may not be limited to morphemes as “minimal meaning bearing units” and morphological structure may also exist within the orthographic level of representation (Longtin et al., 2003; Rastle et al., 2004). In light of this view, the N200 effect likely reflects morphological processing at the orthographic level, given that it occurs early and it can be modulated by orthographic overlap (Zhang et al., 2012).

## Conclusion

In this study, the electrophysiological correlates of morphological processing in Chinese compound reading were investigated with a delayed repetition priming paradigm. Two ERP components including N200 and N400 showed repetition effects that survived across long lags and were interpreted to index morphological processing. There is a possibility that the N200 and its delayed effects reflect general cognitive processing (i.e., low-probability stimulus detection), which needs further testing by manipulating stimulus probability.

### Conflict of interest statement

The authors declare that the research was conducted in the absence of any commercial or financial relationships that could be construed as a potential conflict of interest.
